# Laser-Induced Backward Transfer of Light Reflecting Zinc Patterns on Glass for High Performance Photovoltaic Modules

**DOI:** 10.3390/ma16247538

**Published:** 2023-12-06

**Authors:** Kazimierz Drabczyk, Piotr Sobik, Grażyna Kulesza-Matlak, Olgierd Jeremiasz

**Affiliations:** 1 Institute of Metallurgy and Materials Science, Polish Academy of Sciences, ul. Reymonta 25, 30-059 Kraków, Poland; kazimierz.drabczyk@wp.pl (K.D.); g.kulesza@imim.pl (G.K.-M.); 2 Helioenergia Sp. z o.o., ul. Rybnicka 68, 44-238 Czerwionka-Leszczyny, Poland; piotr.sobik@helioenergia.com

**Keywords:** photovoltaic, PV modules, laser, metal layer, material transfer

## Abstract

Commercially available photovoltaic (PV) modules typically consist of individual silicon half-cut cells that are electrically interconnected. This interconnection method results in gaps between the cells, which do not contribute to the overall PV output power. One approach to enhance the cell-to-module power ratio is the placement of white, diffuse reflecting plastic material within these gaps. Conventionally, the process of generating reflective patterns involves several discrete steps, including film deposition, resist patterning, etching, and resist stripping. This study presents an innovative single-step procedure for the direct deposition of zinc reflective patterns onto glass substrates using laser-induced backward transfer (LIBT) and a nanosecond pulsed laser system. The process successfully produced lines and squares, demonstrating its versatility in achieving diverse geometric patterns under ambient atmospheric pressure and room temperature conditions. The evaluation of the transferred patterns included an examination of geometric dimensions and surface morphology using a 3D microscope and scanning electron microscopy (SEM) analysis at the air/Zn interface. Additionally, the thickness of the zinc film and its adhesion to the glass substrate were quantified. The angular reflectance at a wavelength of 660 nm for both the glass/Zn and air/Zn interfaces was measured.

## 1. Introduction

Commercially available photovoltaic (PV) modules typically consist of separate silicon half-cut cells electrically interconnected, resulting in intercellular gaps that do not contribute to the overall PV module power output. These gaps, though electrically inactive, serve as a noticeable design element for module manufacturers.

In conventional PV systems, the positive electrode of one cell is connected to the negative electrode of the next cell, and this pattern repeats alternately. Copper tapes are used to make these connections, soldering them to the cell electrodes. In earlier module designs, the gap between cells ranged from 2.5 to 3 mm, but in modern commercial PV modules, this gap has been reduced to approximately 1.5 to 2 mm. A 2 mm gap is the most typical [1,2,3].

The choice of a 1.5 mm gap is not arbitrary; narrower gaps were found to cause microcracks on the edges of the cells. These cracks were predominantly observed at the beginning and end of the busbars and tended to propagate along their length [4,5]. Manufacturers, keen on ensuring the absence of such defects, are cautious about further narrowing the intercellular spacing and preserving the flexibility of the connecting tapes they employ.

Nevertheless, manufacturers are actively exploring opportunities to reduce the width of these gaps. LONGI, for instance, introduced an innovative solution: a specialized type of connecting tape that allows gaps to be reduced to 0.6 mm [1]. LONGI’s connecting tape features a novel cross-sectional design, with the part located at the cell’s active surface having a triangular cross-section, while the section bridging the gap between cells and connecting to the rear electrode is significantly flattened. However, it is crucial to note that despite these reductions, the area within the gaps remains optically and thus electrically inactive.

Newer technologies have emerged that enable a shingled layout, eliminating the gaps between solar cells [6,7,8,9,10]. As such, half-cut PV modules with multi-busbar (MBB) technologies, considered mainstream options with high maturity levels, continue to see improvements [11,12].

An alternative approach involves harnessing solar energy incident on the interstitial gaps between photovoltaic (PV) cells. In typical photovoltaic modules, as commonly employed in mainstream applications and notably featured in bi-facial modules [13,14,15], this is accomplished by introducing white, diffusely reflecting plastic material (backsheet) into these gaps. In addition, a white backsheet is advantageous for another reason. Research on the deposition of dirt on PV modules operating in urban conditions has shown that on average, 10–11% more dirt settles on modules with a black backsheet than on modules with a white backsheet [16].

**Figure 1:**
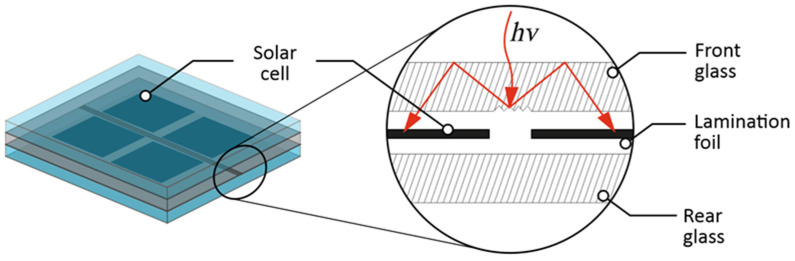
Model of the module with a saw-tooth diffuser placed in the inactive (intercell) space of the module.

More sophisticated methodologies seek to optimize the utilization of these regions by incorporating mirrors that redirect incident solar radiation toward the PV cells. This solution is founded upon the integration of a diffuser within the photovoltaic module. The diffuser is intricately etched into the inactive areas of the module, specifically within the interstices between cells, along the edges, and above the busbars. The texturing of the glass surface is carried out through the application of a CO_2_ laser, which interacts with soda-lime float glass to enable precise surface engraving. The diffuser itself comprises an array of prismatic grooves, featuring both triangular and sawtooth profiles, meticulously incised into the glass surface. Subsequently, the grooved glass is coated with a light-reflecting material [17]. A visual representation of this concept is presented in Figure 1.

Grooving geometry is controlled as described in [17,18] and the saw-tooth profile is designed as a reflector. This saw-tooth profile would work best with normal light reflection. This study introduces a one-step process for depositing zinc reflective patterns directly onto glass substrates using laser-induced backward transfer (LIBT) with a nanosecond pulsed laser system. Lines and squares were successfully fabricated under various laser fluences at ambient atmospheric pressure and room temperature. An evaluation of the transferred patterns included the examination of geometric dimensions and surface morphology using a 3D microscope and scanning electron microscopy (SEM) analysis at the air/Zn interface. Furthermore, measurements were conducted to assess the zinc film thickness and its adhesion to the glass substrate. The angular reflectance at a wavelength of 660 nm was determined for both the glass/Zn and air/Zn interfaces, providing insights into the reflective properties of the deposited patterns.

## 2. Materials and Methods

### 2.1. LIBT Processing

**Figure 2:**
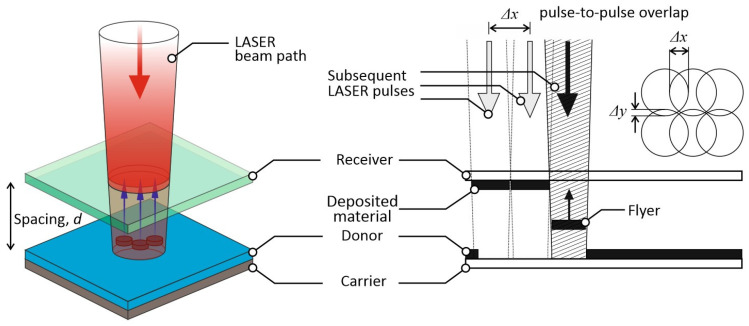
Laser Induced Backward Transfer (LIBT) setup diagram.

LIBT is a technique enabling the transfer printing of a wide range of materials onto a transparent substrate. A very similar technique, called Laser Induced Plasma Assisted Ablation (LIPAA), is also described in the literature [19,20,21,22]. In the LIBT process, the laser beam passes through the transparent receiving glass substrate onto the surface of the donor material, inducing a small amount of donor material to be deposited on the rear of the transparent glass substrate facing the donor material. A nanosecond laser with a wavelength of 1060 nm was used to conduct the LIBT experiments in this study, as shown in Figure 2. The laser beam was deflected with an x–y galvo scanner, passing through the float soda-lime glass and then focused on the surface of the target with an f-theta lens (focal length f = 245 mm). There was no gap between the donor material and the receiver. The laser average power, pulse repetition frequency, hatching distances in the x and y direction, and the scanning speed were controlled using the commercial software EzCad.

### 2.2. Patterning Parameters

**Figure 3:**
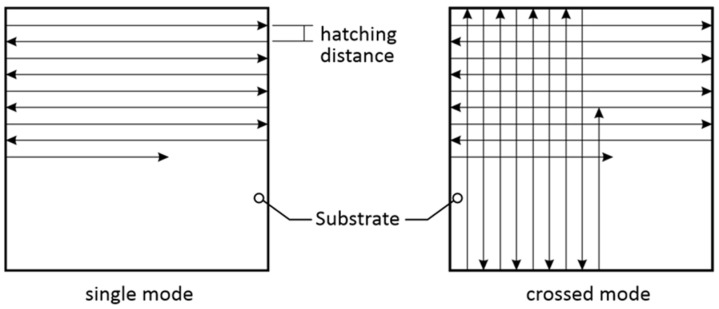
Schematic diagram of hatching patterns. The arrows indicate direction of the laser scan.

The single-line patterns and 40 × 40 mm two-dimensional (2D) square patterns were produced using the LIBT process. Figure 3 illustrates the schematic diagram of the hatching patterns. In this study, the laser fluence varied from 6.1 J/cm^2^ to 39.6 J/cm^2^, and the scanning speed was changed from 600 to 1400 mm/s. For 2D patterning, the hatch distance of the laser scanning path was varied from 50 to 100 µm, and the hatching pattern applied was single and crossed, as shown in Figure 3.

### 2.3. Donor and Receiver Preparation

A bulk solid 1 mm thick sheet of 99% Zn, Cu 0.08 ÷ 0.2%, Ti 0.06 ÷0.1%, Al ≤ 0.015% was used as zinc donor. After each LIBT process, the donor was mechanically abrased to clean and recover the flatness of the donor. Glass substrate was commercial soda-lime float glass 2 or 4 mm thick. The approximate composition was 70% SiO_2_, 15% Na_2_O, 10% CaO, and a few percent MgO, BaO, Al_2_O_3_. The patterns were deposited on the non-tinned side. Before the LIBT process, these substrates were cleaned with 99.5% isopropanol (IPA). After the LIBT process, the substrates were cleaned manually by wiping with a special dust-proof cloth (Sontara DuPonte) and IPA.

### 2.4. Ablation Threshold and Beam Waist Determination

Absolute calibration of laser-energy fluences is essential for quantitative studies of laser interactions with materials. The accuracy of this calibration depends primarily on the measurement of the laser-beam spot size. To determine the ablation threshold and beam waist at 1/e^2^, the method described in [23] is applied. This technique provides a convenient means for determining the exact Gaussian beam spot size at the interaction surface. Precise beam spot-size determination enables pulse-to-pulse overlap degree calculation. The threshold of LIBT process is the minimal laser fluence beginning to induce material transferring from the surface of the donor target material to substrates. Measuring the trench diameter W for different average laser fluences $F_{0}^{av}$ and using the linear relationship between squared trench width *W*^2^ and the natural logarithm of actual fluence $F_{0}^{av}$ to ablation threshold *F_th_* enables for beam radius w_0_ determination.

For a Gaussian spatial beam profile, the radial distribution of the laser fluence is a function of beam radius *w*_0_ and is presented by formula (1):

(1) $$E\left(r\right)=E_{0}^{max}\exp\left(-\frac{2r^{2}}{w_{0}^{2}}\right)$$

and

(2) $$F_{0}^{av}=\frac{E_{pulse}}{\pi \;w_{0}^{2}}$$

*E(r)*—radial distribution of laser energy;
$E_{0}^{max}$—peak laser energy;
$F_{0}^{av}$—average value of fluence;
$E_{pulse}$—total pulse energy.

Solving the above formulas with *r* = *W*/2, and recognizing that *W* = 0 at threshold laser fluence *F_th_*, we obtain:

(3) $$W^{2}=2w_{0}^{2}\ln\left(\frac{F_{0}^{av}}{F_{th}}\right)$$

**Figure 4:**
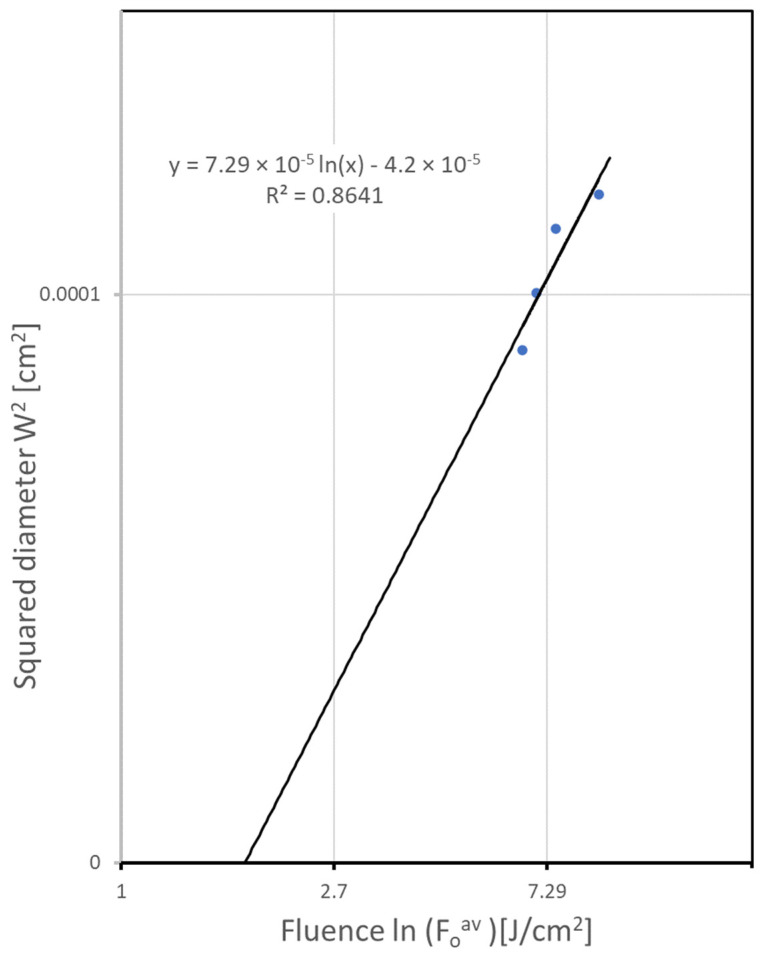
The ablation threshold measurements of zinc plate with 100 ns laser pulses of 1060 nm radiation. The squared diameter *W*^2^ of the ablated area is plotted as a function of the laser fluence $F_{0}^{av}$. The slope of the linear fit (Equation (3)) yields the beam radius at the surface, *w*_0_, and the extrapolation to zero provides the ablation threshold *F_th_*. R^2^ is a coefficient of determination for the linear fit.

The bare donor zinc plate was ablated by laser at different energy levels. This was achieved by varying frequency at constant average power. To calculate the energy, the actual average power was divided by pulse frequency (number of pulses in 1 s). The width of the trench was measured by a digital microscope. The measured values are presented in a semi-log plot in Figure 4:

The extrapolation of the linear fit of Equation (3) to *W*^2^ = 0 results in ablation threshold of *F_th_* = 0.57 J/cm^2^. The beam radius focused on the surface can now be determined by estimating the slope of this linear fit to data points. We obtain a 1/e^2^ Gaussian beam diameter 2*w*_0_ equal to 62.1 µm. Pulse-to-pulse overlap degree in *x* direction Δ*x* can be now defined as a relationship between scan speed and laser pulse frequency:

(4) $$\Delta x=1-\left(\frac{V}{f\;{2w}_{0}}\right)$$

Δ*x*—pulse-to-pulse overlap degree in x direction;
V—scan speed;
f—laser pulse frequency.

Pulse-to-pulse overlap degree in y direction Δ*y* is defined as:

(5) $$\Delta y=1-(\frac{h}{{2w}_{0}})$$

Δ*y*—pulse overlap degree in y direction;
h—hatching line-to-line distance.

By varying laser scan speed and pulse frequency different pulse-to-pulse overlap degree Δ*x* parameters can be achieved. Obviously, different pulse overlap degree in y direction—Δ*y* is determined by hatching line-to-line distance variation.

### 2.5. Research Methods

**Figure 5:**
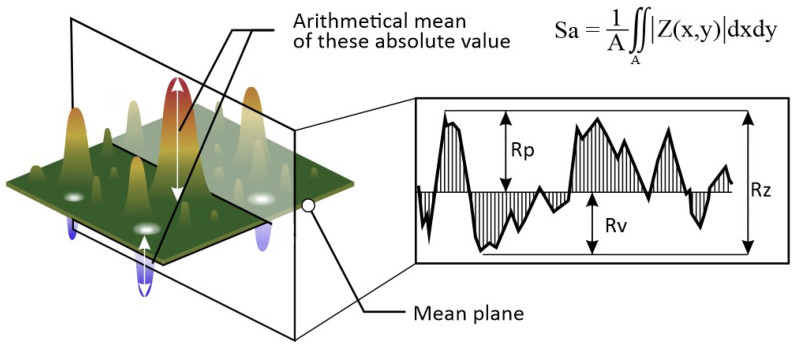
Arithmetic mean height—Ra, Sa and maximum height—Rz, Sz graphical representation. Source: https://www.keyence.com. Accessed on 28 October 2023.

The geometric line width, arithmetic mean height—Ra and maximum height—Rz surface roughness parameters were measured, as well as their areal extensions Sa and Sz. Graphical representation is given in Figure 5. The morphology of deposited patterns on the substrates was measured with a three-dimensional microscope Keyence Corporation, Osaka, Japan, type VHX7000 to investigate micron scale topography of the air/Zn interface and scanning electron microscopy Carl Zeiss AG, Oberkochen, Germany, type LEO 1530 to investigate sub-micrometer morphology of the air/Zn interface.

As indicated in Ref. [18], for a proposed application in PV modules, the zinc layer thickness shall be thin enough to imitate the saw tooth profile shown in Figure 1. Efficient saw-tooth profile height is in a range of 200 µm. The effective reflecting layer shall be in the range of single micrometers to keep the geometry of the saw tooth profile intact. The layer shall also be thick enough to avoid light propagation through the layer (light leakage). The thickness of the obtained layers was measured using X-ray fluorescence method by XRF spectrometer Helmut Fischer, Sindelfingen, Germany, type Fischerscope XDLM 237.

Because the zinc layer is a part of the PV laminated structure, the layer integrity with glass substrate must be assured. Therefore, the adhesion force of the zinc layer to the glass was measured by the method described in ASTM D4541-09 standard [19] using adhesion tester DeFelsko, Ogdensburg, NY, USA, type PosiTest AT-M.

**Figure 6:**
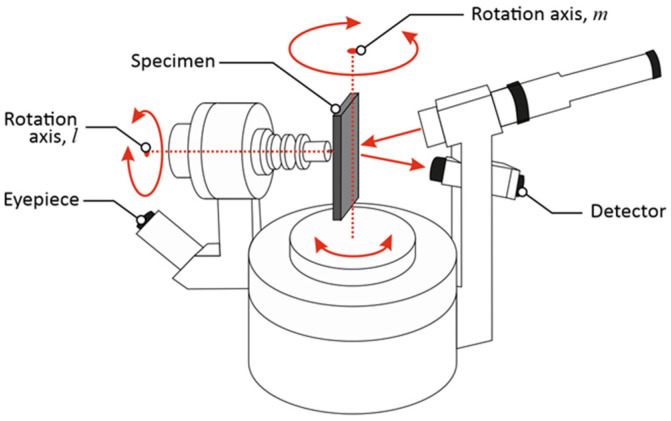
Measurement setup for angular light reflectance measurement.

Efficient light reflectance is necessary to make the solution shown in Figure 1 (red lines) work. Therefore, angular light reflectance was measured using a rotating stage with 30 mW laser source centered at 660 nm and a light power meter United Detector Technology, Hawthorne, CA, USA, type 351. The measurement setup was used as shown in Figure 6.

A commercial aluminum mirror from Thorlabs Inc., Newton, MA, USA with a declared reflectance of 95.68% at 660 nm was used as a reference.

## 3. Results and Discussion

### 3.1. Transferred Line Patterns of Zinc on Glass Substrates

A series of LIBT processes were done on glass samples, which were 4 mm thick. Single lines were transferred.

**Figure 7:**
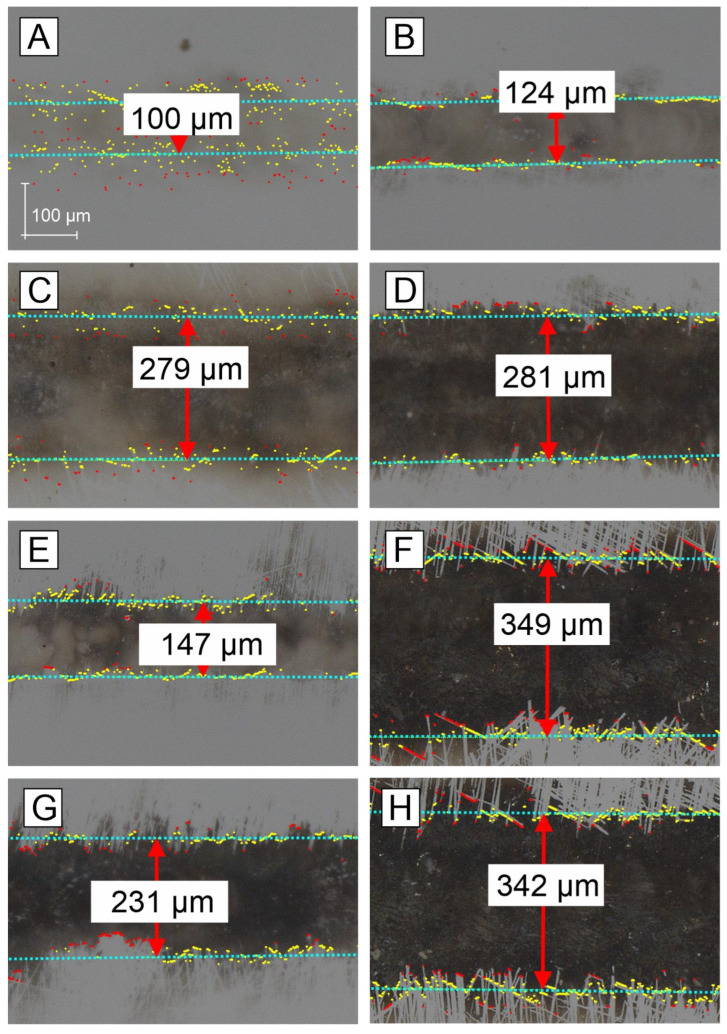
Optical images of transferred lines for different fluences and overlap degree: (**A**)—9.14 J/cm^2^, pulse overlap degree 75%; (**B**)—10.8 J/cm^2^, pulse overlap degree 71%; (**C**)—15.23 J/cm^2^, 75%; (**D**)—18 J/cm^2^, 71%, (**E**)—23.7 J/cm^2^, 36%; (**F**)—22.01 J/cm^2^, 64%; (**G**)—31.7 J/cm^2^, 36%; (**H**)—28.3 J/cm^2^, 54%.

Optical images of transferred lines were obtained from a digital microscope, as shown in Figure 7. Line thickness was measured using the “edge detection” function, which applies the least squares method to automatically detect the edge to be measured, reducing the measurement variation between users.

The minimum fluence that leads to smooth-edged lines is around 15 J/cm^2^. Pulse overlap degree of 64% appears to be a minimum value to obtain lines with well-defined and smooth edges. Figure 7’s comparison of pictures A and B leads to the conclusion that at very low fluences, even a small increase in fluence is effective, as the line becomes much more sharp and visible. C+D pair shows that minor changes in the fluence of 3 J/cm^2^ significantly changes the picture of the line. C+D pair, when compared to A+B pair, shows the same, but indicates that moving from 9–11 J/cm^2^ region to 15–18 J/cm^2^ a line width doubles. If we notice that laser spot size is 62 µm, we see that the ablation plume is very omnidirectional. The E+F pair shows range of fluence 22–23 J/cm^2^ and a difference between very low and high Δ*x* overlap degree. The G-H pair compare low and high Δ*x* overlap degree in high fluence regime. Despite higher fluence picture G shows more narrower line than picture H. Comparing C, E, and G, we see the effect of fluence increase in low Δ*x* overlap regime. Looking at D, F, and H, we observe the effect of change in fluence in high Δ*x* overlap degree.

### 3.2. Transferred Square Patterns of Zinc on Glass Substrates

A series of LIBT experiments were carried out on glass samples 4 mm thick. The 40 × 40 mm patterns were generated with variable laser fluence, power overlap degree, and for single and cross hatch.

**Figure 8:**
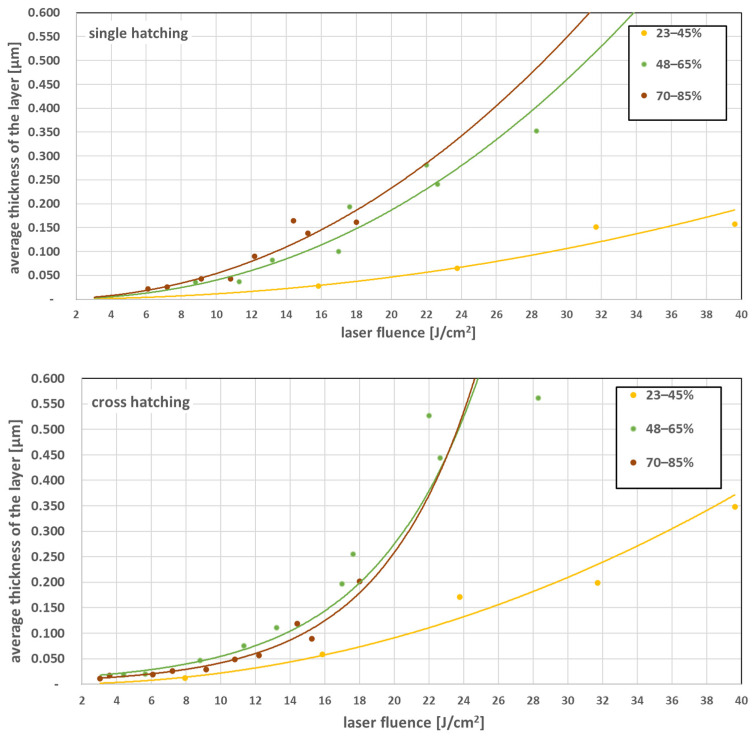
Average thickness of the zinc layer as function of laser fluence for single and crossed hatching pattern, grouped for pulse-to-pulse overlap degree Δ*x* ranges: low: 23–45%, medium: 48–65%, high: 70–85%.

Average zinc layer thickness was measured for different laser fluences and grouped in three pulse-to-pulse overlap degree Δ*x* ranges low: 23–45%, medium: 48–65% and high: 70–85%. Hatching line-to-line distance in y direction was 100 µm resulting in Δ*y* = −61%. Results are summarized in Figure 8.

Medium and high pulse-to-pulse overlap degree Δ*x* 48–85% produce a similar effect in terms of layer thickness vs. fluence. Cross-hatching adds to layer thickness in the fluence range above 20 J/cm^2^ compared to the single hatch pattern. This is interesting to notice. In the cross hatching pattern, the second hatching process is partly counterproductive. The glass substrate is already covered with the first hatching produced zinc layer and the energy of the laser is attenuated, and also the counterproductive of transfer the zinc from glass back to the donor can occur. The conclusion is that a single layer of zinc in a low fluence region does not fully prevent the laser pulse penetration through the layer.

**Figure 9:**
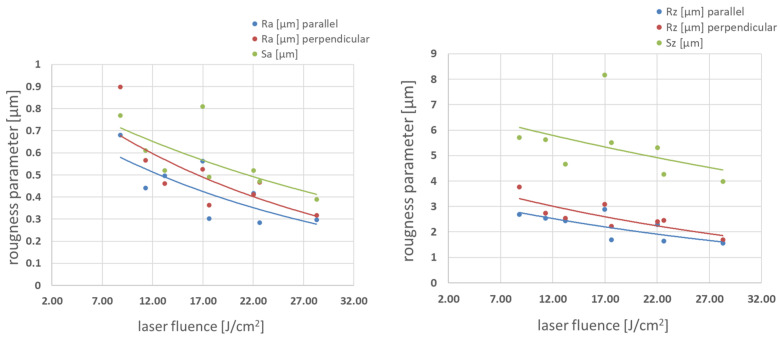
Roughness parameters vs. laser fluence in Δ*x* 54–64% pulse-to-pulse overlap degree range.

**Figure 10:**
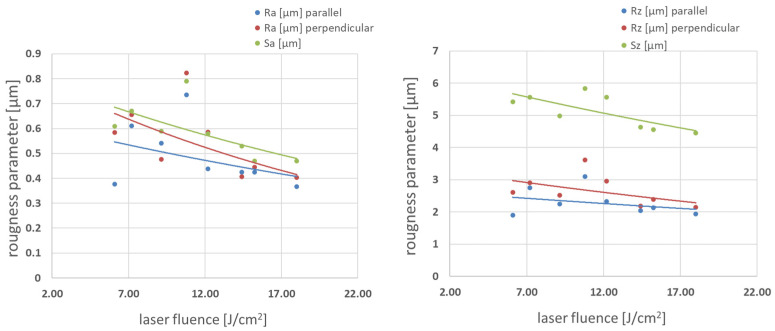
Roughness parameters vs. laser fluence in Δ*x* 71–75% pulse-to-pulse overlap degree range.

Square patterns were examined for profile height deviations (Ra) and maximum peak to valley height of the profile (Rz). Ra and Rz were measured in the direction parallel to the final hatching direction, and the perpendicular was measured across these hatching direction. The corresponding areal parameters within the defined area Sa and Sz were also measured. Results are presented in Figure 9 and Figure 10.

**Figure 11:**
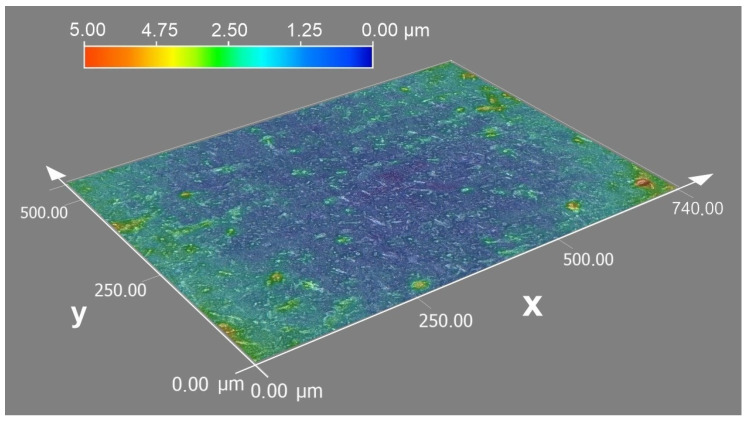
Typical optical microscopy topography visualization of air/Zn interface. Pulse overlap degree Δ*x* = 71%, Δ*y* = 5%. Fluence 18.01 J/cm^2^.

Increasing fluence always leads to lower roughness parameters for both medium and high pulse-to-pulse overlap degrees. Values of Ra and Rz measured in the direction parallel to the final hatching direction are lower than those perpendicular to the hatching direction in all fluence ranges. Optical topography visualization is shown in Figure 11. The topography reveals a smooth surface with low roughness parameters, confirming parameters presented in Figure 9 and Figure 10. Typical glass/Zn interface appears shining, while the air/Zn interface looks grey or even dull black to the naked eye. To find an explanation, SEM imaging was performed.

**Figure 12:**
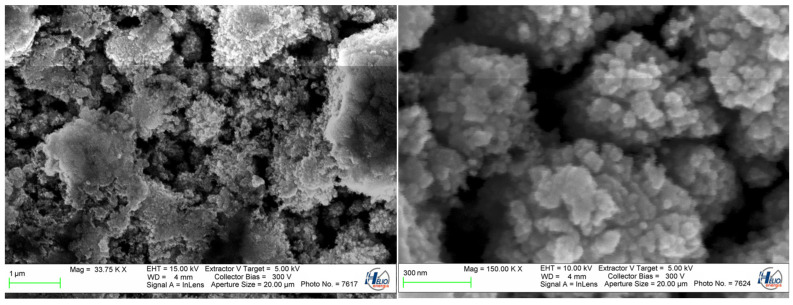
SEM images of air/Zn interface. Pulse overlap degree Δ*x* = 71%, Δ*y* = 5%. Fluence 18.01 J/cm^2^.

SEM imaging revealed the sub-micron structure shown in Figure 12, which is probably responsible for the black visual appearance of the air/Zn interface.

The as received raw air/Zn interface is not useful as a mirror.

### 3.3. Reflectivity Measurement

**Figure 13:**
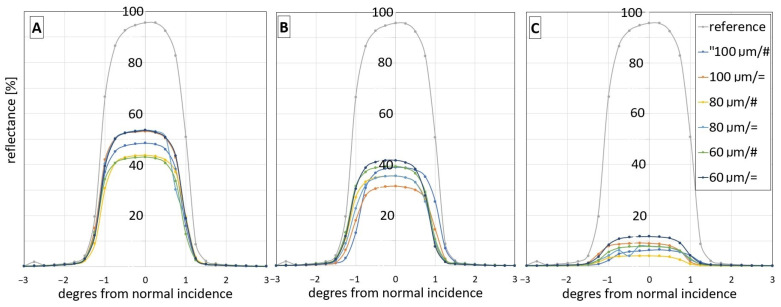
Reflectance for different overlapping line-to-line distance y: 100 µm, 80 µm, 60 µm. “=” stands for single hatching, “#”—stands for cross hatching of: (**A**)—glass/Zn interface at laser fluence of 18 J/cm^2^, pulse overlap degree Δ*x* 71%, (**B**)—glass/Zn interface at laser fluence of 10.8 J/cm^2^ pulse overlap degree Δ*x* 71%, (**C**)—air/Zn interface at laser fluence of 10.8 J/cm^2^ pulse overlap degree Δ*x* 71%.

This study aims to evaluate Zn layers as practical mirror surfaces. Therefore, a series of reflectivity measurements were taken using a 30 mW 660 nm laser source and power meter, using the equipment described in Section 2. The 2 mm thick glass samples were produced with 40 × 40 mm zinc patterns deposited by the LIBT method. The results are presented in Figure 13.

The highest reflectivity is achieved for 18 J/cm^2^ and 71% pulse overlap degree. The best reflectivity achieved is 55% for the glass/Zn interface. A single hatch pattern results in higher reflectivity. Line-to-line distance y in the range 60–100 µm is not influencing reflectance in significant way. Raw air/Zn interface is not useful as a mirror surface, with reflectivity estimated to be 10%. Both interface’s reflection is directional. Diffuse reflection was not detected.

### 3.4. Adhesion Measurement

Because the zinc layer is a part of the PV laminated structure, the layer integrity with glass substrate must be assured. Therefore, adhesion of the zinc layer to glass was measured by the method described in Section 2. The test barrel is glued onto the zinc layer and, after polymerization, the barrel is pulled off and force is measured and registered. Three samples of Zn layer made with fluence 18 J/cm^2^ and Δ*x* 71% pulse overlap degree in cross-hatch pattern were tested.

In all cases, the glue between the barrel and zinc layer was broken first. Minimum adhesion strength is therefore determined to be 20.4 MPa. Any industrial coating adhesion to a substrate above 5.0 MPa is considered as good [24]. Therefore, the achieved result is satisfactory.

## 4. Conclusions

Knowledge of real laser spot size diameter is important as an input to calculate real pulse overlap Δ*x* degree. A non-direct but convenient method described in [23] to determine real laser beam spot size was confirmed as useful in this article. Pulse overlapping Δ*x* is the most critical parameter in LIBT. By analyzing line width and regularity, it can be found that effective pulse-to-pulse overlap degree lies in the medium range of overlapping 54% to 64%. Results of experiments with producing the square patterns show both fluence and pulse overlap Δ*x* degree influence on layer thickness. Pulse overlap degree Δ*x* in the range 70–85% in single-hatch pattern produces the highest layer thickness, which is only fluence dependent. In cross-hatch, 48–85% pulse overlap Δ*x* produced a similar thickness, also fluence dependent. Applying single hatching instead of cross-hatching increases the layer thickness in the low overlap degree range and high fluences above 20 J/cm^2^.

An important notice is that, despite some increase in layer thickness, the cross-hatching is not increasing reflectivity. Fluence around 30 J/cm^2^ leads to a layer thickness of 0.6 µm. Such layer thickness is still leaky for the light, and enables for light reflection of 55%. Reflectivity can be further increased based on conclusions from this study. Because of the laser system, the power limitation used in the experiment the fluence level was also limited. Higher fluence most probably will increase layer thickness and decrease light leakage, thus increasing reflectivity. A two-step process with a cross-hatching pattern can be further studied. The first hatch with low fluence followed by a high fluence hatch can be the solution. Pulse-to-pulse overlap degree Δ*x* must be controlled carefully. The line-to-line distance in “*y*” direction in the range 60–100 µm does not influence reflectivity, and is considered less important. The air/Zn interface in raw form is not an effective mirror. For bifacial PV module architecture, a separate step of polishing the metal layer must be considered. The Zn layer adhesion of 20.4 MPa to glass is high, and suitable for the PV module lamination process. A side effect of the study is that the air/Zn surface has an expanded sub-micron surface morphology, which can be further studied for other purposes.

## Data Availability

Data are contained within the article.
